# Supplementary material for: Resolving the full spectrum of human genome variation using Linked-Reads
Source: Genome Res. 2019 Apr;29(4):635–45. doi: 10.1101/gr.234443.118 (PMC6442396; doi:10.1101/gr.234443.118)
Supplement: Supplemental Material [file supp_gr.234443.118_Supplemental_longranger-2.2.zip › longranger-2.2.tar.bz2!/lr2.2-public/lib/stan/cmdstan/stan/doc/api/html/fwd_2functions_2fma_8hpp.html]

Stan: src/stan/agrad/fwd/functions/fma.hpp File Reference


|  |  |
| --- | --- |
|  | Stan  2.5.0  probability, sampling & optimization |


- Main Page
- Related Pages
- Namespaces
- Classes
- Files

- File List
- File Members

All Classes Namespaces Files Functions Variables Typedefs Enumerations Enumerator Friends Macros Pages

- src
- stan
- agrad
- fwd
- functions

Namespaces |
Functions

fma.hpp File Reference

`#include <cmath>`  
`#include <stan/agrad/fwd/fvar.hpp>`  
`#include <stan/meta/traits.hpp>`

Go to the source code of this file.

|  |  |
| --- | --- |
| Namespaces | |
|  | stan |
|  | Probability, optimization and sampling library. |
|  | |
|  | stan::agrad |
|  | Function gradients via reverse-mode automatic differentiation. |
|  | |

|  |  |
| --- | --- |
| Functions | |
| template<typename T1 , typename T2 , typename T3 > | |
| fvar< typename   stan::return\_type< T1, T2, T3 >  ::type > | stan::agrad::fma (const fvar< T1 > &x1, const fvar< T2 > &x2, const fvar< T3 > &x3) |
|  | The fused multiply-add operation (C99). More... |
|  | |
| template<typename T1 , typename T2 , typename T3 > | |
| fvar< typename   stan::return\_type< T1, T2, T3 >  ::type > | stan::agrad::fma (const T1 &x1, const fvar< T2 > &x2, const fvar< T3 > &x3) |
|  | See all-var input signature for details on the function and derivatives. More... |
|  | |
| template<typename T1 , typename T2 , typename T3 > | |
| fvar< typename   stan::return\_type< T1, T2, T3 >  ::type > | stan::agrad::fma (const fvar< T1 > &x1, const T2 &x2, const fvar< T3 > &x3) |
|  | See all-var input signature for details on the function and derivatives. More... |
|  | |
| template<typename T1 , typename T2 , typename T3 > | |
| fvar< typename   stan::return\_type< T1, T2, T3 >  ::type > | stan::agrad::fma (const fvar< T1 > &x1, const fvar< T2 > &x2, const T3 &x3) |
|  | See all-var input signature for details on the function and derivatives. More... |
|  | |
| template<typename T1 , typename T2 , typename T3 > | |
| fvar< typename   stan::return\_type< T1, T2, T3 >  ::type > | stan::agrad::fma (const T1 &x1, const T2 &x2, const fvar< T3 > &x3) |
|  | See all-var input signature for details on the function and derivatives. More... |
|  | |
| template<typename T1 , typename T2 , typename T3 > | |
| fvar< typename   stan::return\_type< T1, T2, T3 >  ::type > | stan::agrad::fma (const fvar< T1 > &x1, const T2 &x2, const T3 &x3) |
|  | See all-var input signature for details on the function and derivatives. More... |
|  | |
| template<typename T1 , typename T2 , typename T3 > | |
| fvar< typename   stan::return\_type< T1, T2, T3 >  ::type > | stan::agrad::fma (const T1 &x1, const fvar< T2 > &x2, const T3 &x3) |
|  | See all-var input signature for details on the function and derivatives. More... |
|  | |


---

[ Stan Home Page ]

*© 2011–2014,
Stan Development Team.*
